# Inoculability of Diphtheritis

**Published:** 1860-03

**Authors:** 


INOCULABILITY OF DIPHTHERITIS.—In the *Gazette Hebdomadaire*, we find the substance of a discussion at the Medical Society of the Hospitals of Paris, upon the transmissibility of diphtheritis by inoculation, and the report of two cases supposed to have been produced by dissection wounds.

The subject of the first case was a physician, who pricked his finger with a bistoury that had been used in the operation of tracheotomy for croup. In a few hours the wounded part became swollen, and the seat of severe pain, which was followed by the development of an abscess. Fifteen days after the accident, the finger not being completely cured, he exposed himself to cold, and was seized with a chill in the evening, and with a pain in the throat during the night. The next morning false membrane was found on the right tonsil, which, on the following day, had extended to the left gland. Three or four days later his wife was attacked with pseudo-membranous inflammation of the throat, but both recovered.

The second case occurred in the person of a medical student, who pricked his left thumb in making an examination of a child dead of diphtheritis. At the time of the autopsy he had a cough and influenza.

In spite of every precaution, he had on the same evening every symptom of inflammation of the lymphatics, which extended on the following morning to the axilla. On the third day he suffered from sore-throat, and the submaxillary lymphatic glands were slightly enlarged. On the following day the lymphatitis had disappeared, but the angina increased and became pseudo-membranous two days later. On the seventh day the lower lip was attacked with herpes, but in nine days more he was perfectly well.

The majority of the members of the Society did not consider these as true examples of inoculated diphtheritis, but that they were due to epidemic influence. A fact in confirmation of their opinion is, that M. Peter, a resident of the Children's Hospital, has made several unsuccessful attempts to inoculate himself with the matter of membranous croup, by introducing it into his lower lip and even brushing it on his pharynx, without any local or general result.

